# PON1 and PON3 in Alzheimer’s Disease: Similar Functions but Different Roles

**DOI:** 10.3390/antiox13101216

**Published:** 2024-10-10

**Authors:** Alessandro Trentini, Valentina Rosta, Raffaella Riccetti, Gianmarco Mola, Riccardo Galletti, Marco Pinotti, Vincenza Senia, Giovanni Zuliani, Carlo Cervellati

**Affiliations:** 1 Department of Environmental and Prevention Sciences, University of Ferrara, Via Luigi Borsari 46, 44121 Ferrara, Italy; valentina.rosta@unife.it (V.R.); raffaella.riccetti@unife.it (R.R.); 2 Department of Translational Medicine and for Romagna, University of Ferrara, Via Luigi Borsari 46, 44121 Ferrara, Italy; gianmarco.mola@unife.it (G.M.); riccardo.galletti@unife.it (R.G.); marco.pinotti@unife.it (M.P.); vincenza.senia@unife.it (V.S.); zlngnn@unife.it (G.Z.)

**Keywords:** Alzheimer’s disease, paraoxonase, mild cognitive impairment, high-density lipoprotein

## Abstract

Paraoxonase 1 (PON1) and Paraoxonase 3 (PON3) are enzymes located on the surface of high-density lipoprotein (HDL) and share similar antioxidant properties, possibly modulated by other proteins such as Myeloperoxidase (MPO), which drives the shift from functional to dysfunctional HDL. PON1 has been extensively studied in relation to Alzheimer’s Disease (AD), but the role of PON3 remains unknown. To fill this knowledge gap, the study analyzed PON3 protein levels and PON1-arylesterase activity in 99 AD patients, 100 patients with mild cognitive impairment (MCI), and 79 cognitively normal controls. The results showed that serum PON3 levels remained unchanged across all groups. In contrast, serum arylesterase activity was significantly reduced in both AD and MCI patients compared to controls (*p* < 0.001 for both comparisons). Surprisingly, there was no correlation between arylesterase activity and MPO protein concentration or activity. However, PON3 was found to have a significant positive correlation with both MPO concentration (r = 0.507, *p* < 0.0001) and MPO activity (r = 0.264, *p* < 0.01). In conclusion, we demonstrated for the first time that PON1 and PON3 have distinct relationships with AD, with only PON1 showing a decrease in activity in this disease, while PON3 levels remained unchanged. Another noteworthy finding was the selective correlation between PON3 and MPO, which may suggest a preferential physical association of PON3 with dysfunctional HDL.

## 1. Introduction

Paraoxonases (PONs) are a family of three enzymes, PON1-3, with primarily lactone hydrolyzing activity of still not completely deciphered biological function [1,2,3]. Despite the gap in knowledge, evidence from pre-clinical and clinical studies is consistent with the overall protective role of all three isoenzymes [4]. This protection appears to be mostly exerted by counteracting the harmful lipoperoxidation, especially in low-density lipoprotein, (LDL), uncontrolled inflammation, and the noxious effect of some man-made compounds and their metabolites [2,5].

While PON2 is exclusively located within cells, PON1 and PON3 are predominant within the circulation associated with lipoproteins, particularly high-density lipoprotein (HDL) [4,6,7]. A body of convergent evidence suggests that the well-established anti-atherosclerotic effect of HDL is largely attributable to PON1 [8,9,10,11]. Indeed, PON1 activity was found to be reduced, irrespective of HDL-cholesterol (HDL-C) concentration, in several forms of cardiovascular diseases [12,13], but also in other diseases, including those affecting the brain [5,14,15]. We were among the first to demonstrate a significant reduction in serum PON1 activity in patients with Alzheimer’s disease (AD) and Vascular Dementia, the most common forms of dementia [16,17]. Intriguingly, a decrease in PON1 was already observed in patients with the so-called Mild Cognitive Impairment (MCI), the prodromal phase of dementia [18].

Compared to PON1, the literature on PON3 is much more limited. The reasons are two-fold: firstly, the levels of PON3 in plasma are around 100 times lower than those of PON1 [2,3,19,20] making ELISA the preferred technique for its specific quantification. Secondly, an alternative and less expensive method to quantify PON1 in plasma/serum, but not PON3, is via its arylesterase activity. Of note, the activity of PON1 can also be measured by evaluating its paraoxonase (using paraoxon as a substrate) and lactonase activities [19,20]. The latter is widely considered the physiological function of PON1, as it degrades lipolactones in oxidized lipoproteins and cell membranes [3]. As with PON1, PON3 also shows the ability to prevent the oxidation of LDL in vivo [4,21]. However, both lactonase and paraoxonase activities are significantly influenced by common PON1 polymorphisms [19,22]. In contrast, arylesterase activity, despite being a promiscuous activity, serves as a superior surrogate for PON1 concentration due to its minimal interindividual variability and less interference by polymorphisms compared to other measures [19,20].

In vitro studies clearly indicate that both PON1 and PON3 contribute to HDL’s well-established atheroprotective function [11,23]. However, other accessory proteins associated with HDL, particularly Myeloperoxidase (MPO), can negatively impact this beneficial function by inhibiting PON1 activity through oxidative modification [24]. The physical association of MPO with HDL primarily occurs under conditions of uncontrolled inflammation and can trigger a shift from anti-atherogenic to pro-atherogenic, leading to the formation of dysfunctional HDL [25,26]. This transition undermines HDL’s protective role and promotes atherogenesis [25]. Unlike PON1, the interaction between MPO and PON3 has never been documented.

Since the arylesterase and lactonase activities of PON3 are significantly lower than those of PON1 (and paraoxonase activity is absent in PON3), the best way to assess PON3 is by detecting its protein amount. Pursuing this approach, Salazar et al. found that the expression of both PON1 and PON3 is altered in the brain areas of a mouse model of Alzheimer’s disease with abundant Amyloid β (Aβ) plaques [27].

Despite the potential significance of PON3 in Alzheimer’s disease pathology and its functional similarities to the extensively studied PON1, no human studies have investigated whether changes in peripheral PON3 levels are associated with the disease. To address this gap, we measured the serum concentration of PON3, along with arylesterase activity, in a sample consisting of cognitively normal controls, as well as individuals with MCI and AD.

## 2. Materials and Methods

### 2.1. Subjects

This study examined 278 subjects, a subset of a cohort previously used to assess arylesterase activity in AD and controls [18], along with a new group of MCI. The study population included:

Ninety-nine subjects with mild/moderate probable late-onset AD were diagnosed by the National Institute on Aging and Alzheimer’s Association (NIA-AA) workgroups criteria [28]. Standardized Mini-Mental State Examination (MMSE) [29] range: 18–23.

One hundred MCI patients. Amnestic MCI was defined as the presence of a documented deficit in memory and/or other cognitive domains, without (single domain) or with (multiple domain) impairment in other cognitive domains, in an individual who did not meet the clinical criteria for dementia [30].

Seventy-nine cognitively normal subjects (Controls).

These outpatients were referred to the Memory Clinic of the Department of Internal Medicine, S. Anna University Hospital, Ferrara (Italy)

Exclusion criteria were as follows: age < 65 years; any unstable or severe medical condition (e.g., severe hepatic or renal disease, current cardiovascular disease, cancer); major psychiatric disorders such as uncontrolled depression and schizophrenia; use of non-steroidal anti-inflammatory drugs (or steroids). Personal and medical history data were gathered during the initial visit through a structured interview conducted by trained staff, addressing patients or their caregivers. The criteria for diagnosing diabetes, hypertension, and coronary heart disease (including coronary heart disease, stroke, and smoking classification with a history of smoking 10 or more pack-years) are detailed in another source [18]. General and neuropsychiatric assessments, including the standardized MMSE evaluations of basic activities of daily living (BADL) and instrumental activities of daily living (IADL), and the 15-item geriatric depression scale (GDS) [31], were conducted as described in other publications. Routine clinical chemistry analyses, including serum B-12 vitamin and folate levels, liver, kidney, and thyroid function tests, blood cell count, and arterial oxygen saturation, were performed to rule out secondary causes of cognitive impairment. Additionally, as part of the diagnostic algorithm, all subjects with cognitive impairment or dementia underwent brain Magnetic Resonance Imaging (MRI) and/or 18F-Fluorodeoxyglucose Positron Emission Tomography (18F-FDG-PET) when necessary. The study was approved by the Local Ethic Committee (code number: 170579). All patients were informed about the research project and research protocol. Written consent for research was obtained. The research protocol did not alter the routine clinical or diagnostic procedures used for diagnosing cognitive impairment or dementia, nor did it influence any treatment decisions for the enrolled individuals. This study was conducted in accordance with the World Medical Association’s Code of Ethics (Declaration of Helsinki).

### 2.2. Assessment of Blood Parameters

Peripheral blood samples were obtained via venipuncture into Vacutainer^®^ tubes without anticoagulant following an overnight fast. After a 30 min incubation at room temperature, the samples were centrifuged at 4650× *g* for 20 min. The resulting sera were then collected, divided into single-use aliquots, and stored at −80 °C until analysis. With the exception of lipid profile and glycemia, all the following biochemical parameters were assessed by the spectrofluorometer Tecan Infinite M200 (Tecan Group Ltd., Männedorf, Switzerland).

#### 2.2.1. Lipid Profile and Glycemia

The levels of plasma lipids (total cholesterol, HDL-C, and triglycerides), and glucose were assessed by the centralized laboratory of Sant’Anna Hospital (Ferrara) by standard enzymatic techniques [32]. Levels of low-density lipoprotein (LDL)-cholesterol (LDL-C) were obtained according to Friedewald’s formula [33].

#### 2.2.2. MPO Protein Concentration

The concentration of total MPO was determined by a commercially available Enzyme-Linked Immunosorbent Assay (ELISA) kit by following the manufacturer’s instructions (Human MPO, Boster, Cat. No. EK0850) as described elsewhere [34].

#### 2.2.3. MPO Activity

The MPO activity assay was conducted following the protocol established in our previous study [34]. In summary, 100 μL of anti-MPO polyclonal antibodies (Calbiochem, Cat. No. 475915) were used to coat the wells of an ELISA microplate, and the plate was incubated overnight at 4 °C. Following incubation, the wells were washed three times with 300 μL/well of wash buffer (WB: 0.15 M NaCl, 0.1 M NaH_2_PO_4_, pH 7.2, 0.05% Tween-20) before blocking with 300 μL of 5% Bovine Serum Albumin (BSA) in WB for 1 h at room temperature. After three additional washes with 300 μL/well of WB, 10 μL of serum (diluted 1:5) or standard (ranging from 0.39 to 25 ng/mL MPO purified from leukocytes, Calbiochem, Cat. No. 475911) diluted in 1% BSA in WB were added in duplicate to the wells. The plate was then incubated for 1 h at room temperature with gentle agitation, followed by four washes with 300 μL/well of WB. Each well was subsequently treated with 50 μL of 392 μM H_2_O_2_ (resulting in a final concentration of 196 μM) and 50 μL of 200 μM AmpliFlu Red (Sigma-Aldrich, Cat. No. 90101), both diluted in 20 mM citrate buffer (pH 6) containing 80 mM NaBr. The resulting fluorescence (resorufin) was measured at an excitation wavelength of 535 nm and an emission wavelength of 590 nm every 30 s for 10 min at 37 °C. The fluorescence readings were converted into enzyme Units (U)/L of active enzyme using a standard curve generated from various concentrations of resorufin as previously described [34].

#### 2.2.4. Arylesterase Activity

Arylesterase activity was measured by adding 10 μL of serum, diluted 24 times, to 240 μL of a reaction mixture containing 1 mmol/L phenylacetate and 0.9 mmol/L CaCl_2_ dissolved in 9 mmol/L Tris-HCl at pH 8 [17,35]. The enzyme activity was calculated using a molar extinction coefficient of 1.3 × 10^3^ M^−1^ cm^−1^ and expressed in kilo units per liter. One unit of arylesterase activity corresponds to the production of 1 µmol of phenol per minute under the conditions of the assay. The intra-assay coefficient of variation (CV) was 3.8%, while the inter-assay CV was 9.7%

#### 2.2.5. PON3 Serum Levels Determination

To evaluate serum levels of PON-3, a commercial ELISA kit (elabscience E-EL-H2374) was used. The assay was performed by following the manufacturer’s instructions. This kit operates on the Sandwich-ELISA principle. Briefly, 100 µL of serum samples diluted 10 times, or the standard within a working range 0.23–15 ng/mL, were added to a plate pre-coated with a specific antibody for human PON3. After 90 min of incubation at 37 °C, 100 µL of biotinylated detection antibody specific for human PON3 were added to each well, followed by an incubation of 60 min at 37 °C. At the end of the incubation, three washing steps were performed, each with 300 µL of washing buffer per well, and 100 µL of avidin-horseradish peroxidase (HRP) complex diluted in the appropriate diluent was added to each well. The plate was incubated for 30 min at 37 °C and then washed five times with 300 µL/well of wash buffer. Finally, 90 µL of substrate reagent was added, and after a 15 min incubation period, the reaction was stopped by adding 50 µL of stop solution. The optical density (OD) was measured spectrophotometrically at a wavelength of 450 nm. The amount of PON3 present in samples was determined by interpolation with the standard curve.

### 2.3. Statistical Analysis

Continuous variables were tested for normality using the Kolmogorov–Smirnov and Shapiro–Wilk tests. Normally distributed variables were presented as mean ± standard deviation, and comparisons between groups were performed using analysis of variance (ANOVA), with SIDAK as the post hoc test. Analysis of covariance (ANCOVA) was conducted to check whether the differences detected at univariate analysis remained significant after adjusting for covariates. For variables that did not pass the normality test, values were expressed as median and interquartile range, and differences between groups were assessed using the Kruskal–Wallis test followed by the Mann–Whitney U test for pairwise comparisons, with Bonferroni correction for multiple comparisons. Categorical variables were compared using the Chi-square (χ^2^) test. Comparisons of continuous variables between two groups were made using the t-test for normally distributed variables and the Mann–Whitney U test for non-normally distributed variables. Correlations were estimated using Pearson’s correlation for normally distributed variables and Spearman’s rank correlation for non-normally distributed variables. Multiple linear regression analysis was conducted to determine the independence of the detected correlations. A two-sided *p*-value of <0.05 was considered statistically significant. Statistical analyses were conducted using the SPSS package for Windows (Version 20.0, IBM SPSS, Markham, ON, Canada).

## 3. Results

### 3.1. Main Characteristics of the Population

The study involved 278 older individuals, including both controls and patients with AD and MCI (Table 1). On average, the controls were 2 years younger than the participants in the other two groups (*p* < 0.05 for both comparisons). There was a significantly higher prevalence of females in the AD group compared to both MCI and controls (*p* < 0.01 for both comparisons). As expected, formal education levels were lower in all patient groups compared to controls (*p* < 0.01 for all). Moreover, MMSE scores were higher in controls compared to both the MCI (*p* < 0.01) and AD (*p* < 0.001) groups. Concerning comorbidities, the prevalence of hypertension was significantly higher in MCI relative to the other two groups (*p* < 0.05 for both). On the contrary, no significant between-group difference was detected for Diabetes, coronary heart disease (CHD), stroke and smoking status. No significant differences were detected for lipid profile parameters, plasma glucose, and serum concentration of MPO (see Supplementary Table S1 for MPO values).

**Figure 1:**
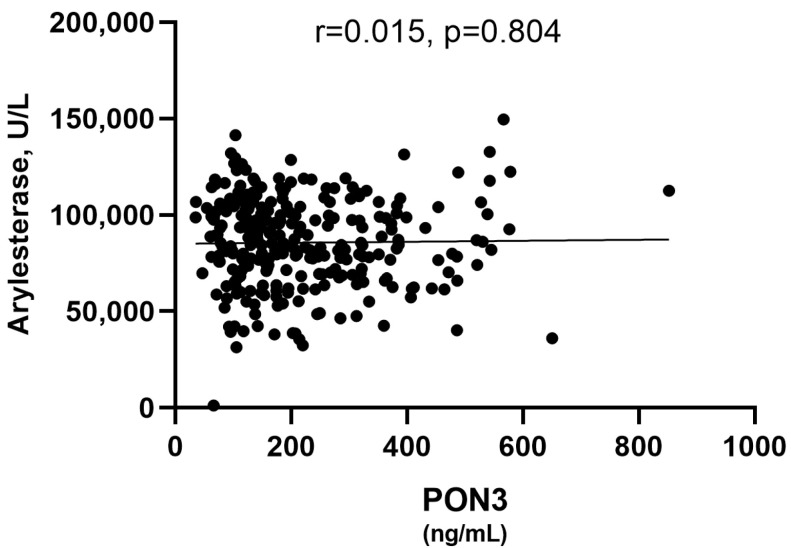
Scatter plot for the analysis of the correlation between serum PON3 concentration and serum arylesterase activity.

Since, to the best of our knowledge, the only study on humans [3] showed that recombinant PON3 is able to catalyze the hydrolysis of phenyl-acetate, although, to a significantly lesser extent compared to PON1, we examined the correlation between the serum concentration of this protein and arylesterase activity. We found that these two variables did not correlate with each other (Figure 1). This finding suggests that the observed arylesterase activity may be solely attributable to PON1.

### 3.2. Evaluation of the Possible Contribution of PON3 to Arylesterase Activity

**Figure 2:**
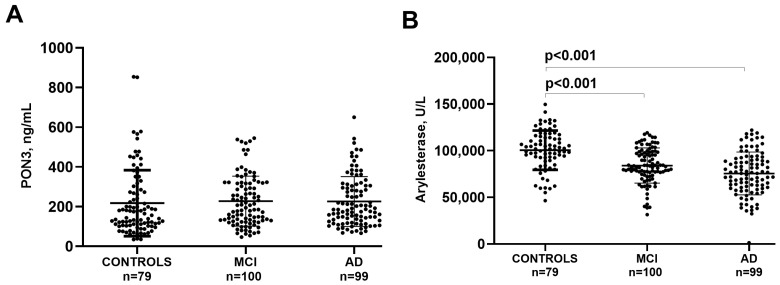
Serum levels of PON3 protein concentration (**A**) and arylesterase activity (**B**) in Controls, MCI and AD. Abbreviations: MCI, Mild Cognitive Impairment; AD, Alzheimer’s disease.

Once we established that serum PON3 protein concentration and arylesterase activity were two distinct metrics, we analyzed their behavior in Controls, MCI and AD. As displayed in Figure 2A, PON3 did not significantly change in Controls, MCI and AD (mean values: 217 ng/mL, 227 ng/mL and 225 ng/mL, respectively). Conversely, arylesterase activity was approximately 16% and 25% lower in MCI (mean ± standard deviation: 84,099 ± 18,846 U/L) and in AD (75,579 ± 22,957), respectively, compared to controls (100,589 ± 21,073) (Figure 2B). Of note, the changes observed for serum arylesterase activity were independent of potential confounding factors such as age, sex and comorbidities (ANCOVA, *p* < 0.001).

### 3.3. Serum Arylesterase Activity and Serum PON3 Concentration in Controls, MCI and AD, and Association of These Markers with Cognitive Status and Brain Atrophy

**Figure 3:**
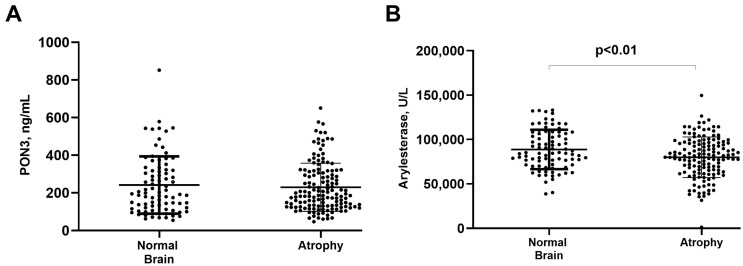
Serum PON3 protein concentration (**A**) and serum arylesterase activity (**B**) in individuals with and without brain atrophy.

The possible association of serum arylesterase activity and PON3 concentration with cognitive and functional status parameters was also evaluated. Arylesterase, but not PON3, was found to have a significant positive correlation with MMSE (r = 0.308, *p* < 0.001), confirming previous findings [2]. Interestingly, lower arylesterase activity, but not PON3, was found in subjects with brain atrophy compared to those not presenting this AD pathological trait (*p* < 0.01, Figure 3B). This difference in arylesterase activity retained its significance after adjustment for age, sex, and comorbidities (*p* < 0.01).

### 3.4. Relationship of PON3 and Arylesterase Activity with Demographic, Clinical and Biochemical Variables

**Figure 4:**
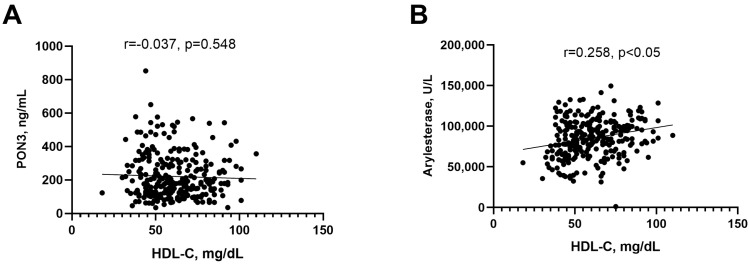
Scatter plot for the analysis of correlation between HDL-C and serum PON3 (**A**) and between serum HDL-C and serum arylesterase activity (**B**).

We then examined whether serum PON3 concentration correlated with commonly identified predictors of arylesterase activity, such as age, sex, and hypertension (correlations between arylesterase activity and these variables were also observed in this study but are not presented herein). No significant correlations were detected between PON3 and these covariates. Similar negative results were obtained when analyzing the correlation between PON3 and HDL-C (Figure 4A). As expected, arylesterase activity was associated with HDL-C (r = 0.258, *p* < 0.05, R^2^ = 0.055 Figure 4B).

### 3.5. Relationship of PON3 and Arylesterase Activity with MPO

**Figure 5:**
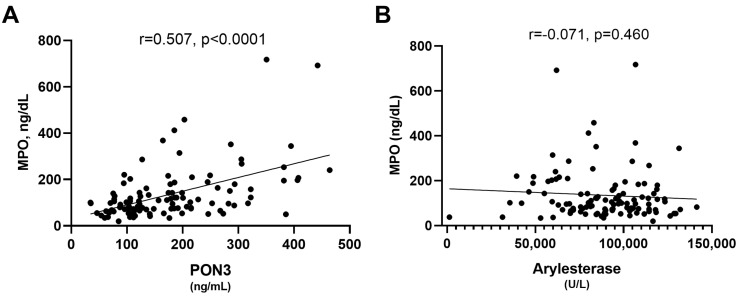
Scatter plot for the analysis of correlation between serum MPO protein concentration and serum PON3 (**A**) and between serum MPO protein concentration and serum arylesterase activity (**B**).

MPO is a well-established determinant of HDL function, along with PON1 and PON3 [24,26]. It was suggested that the pro-oxidant activity of MPO and the antioxidant activity of PON1 influence each other. Given the functional similarities between the two PON isoenzymes, we hypothesized a similar mutual influence for PON3 [24,26]. The analysis revealed that only PON3 exhibited a significant positive correlation with MPO concentration (r = 0.507, *p* < 0.0001, R^2^ = 0.257, Figure 5A). A similar trend was observed with MPO activity, though the association was weaker compared to that with MPO concentration but remained significant (r = 0.264, *p* < 0.01, R^2^ = 0.07, Supplementary Table S1). The strength of these associations remained unaffected after adjusting for potential confounders, such as sex, age, HDL-C and comorbidities. Interestingly, the ratio between MPO concentration and MPO activity, which reflects the enzyme’s specific activity, did not show a significant correlation with PON3. In other words, PON3 did not have any effects on the activity of each MPO particle. Median levels of MPO concentration and activity across the study groups are displayed in Supplementary Table S1.

## 4. Discussion

Our knowledge of PON proteins is largely based on studies centered around PON1. Although the exact physiological role of PON1 has not been fully elucidated, it is widely accepted that its primary biological function in vivo is to prevent the lipid peroxidation of LDL and macrophage membranes, both of which may contribute to accelerated atherosclerosis [5]. In contrast, our understanding of PON3 biology and its role in diseases is limited.

The primary objective of this study was to investigate for the first time the behavior of serum PON3 in patients with AD or MCI. The research is primarily justified by three main sets of findings: (1) Serum PON1 has been widely found to be altered in neurodegenerative diseases, including AD, and this was attributed to the antioxidant capacity of the protein [16,18,36,37,38,39]; (2) both PON1 and PON3 protect LDL from oxidative modification, with the latter being more efficient in this biological activity [1,3]; (3) both PON1 and PON3 are altered in the brain of an AD mice model [27].

Before addressing the main goal of the study, we first investigated whether PON3 could contribute to the observed arylesterase activity since information on this aspect is limited. To the best of our knowledge, the only study comparing lactonase, arylesterase, and paraoxonase activity among the three PON isoenzymes is the one from Draganov et al. [3]. Their findings showed that PON3 possesses the most limited arylesterase activity, approximately 200 times lower than that of PON1 [3]. Moreover, these assessments were conducted using recombinant PON3, which was not associated with HDL, placing it in a different environment than the protein found in serum. Notably, the same study indicated that recombinant PON1 and PON3 are biologically dysfunctional and cannot protect LDL against copper-ion-induced oxidation [3]. In other words, the kinetic data obtained from recombinant PON3 (and PON1) may not fully replicate those obtained from ex vivo measurements. Given these considerations, our finding of a lack of correlation between PON3 and arylesterase activity in the serum of the study subjects suggests that this activity may be solely attributable to PON1. Other studies are in line with our findings [21].

As a confirmation, we found that PON3 did not follow the decreasing trend of the arylesterase activity observed in MCI and AD subjects compared to controls (a trend that is in line with previously published data). The selective association between arylesterase activity and AD was also indicated by its specific correlation with characteristic and progressive changes in the disease, such as cognitive decline (as assessed by MMSE) and cortical atrophy [40,41]. These findings suggest that a decrease in PON1 activity may be linked to the occurrence and progression of AD pathology.

The observation that PON3 remains unaltered in patients with AD and MCI can be interpreted in several ways. The most straightforward explanation might be that the antioxidative effect of PON3 on LDL does not significantly impact AD pathogenesis. The ability of PON3 to exert this antioxidative action is one of the few known aspects of its biological and physiological functions. In addition, the narrower substrate specificity of PON3, compared to PON1, may limit its role in diseases, including AD. Indeed, unlike PON1, PON3 lacks organophosphatase activity against synthetic substrates like paraoxon, chlorpyrifos oxon, and diazoxon [3], making it ineffective at protecting against the harmful effects of environmental toxicants, which have been extensively linked to AD [42,43]. In addition, the aforementioned study on recombinant PONs showed that with the sole exception of lovastatin and spironolactone, the affinity of PON3 for lactones is generally lower than that of PON1 [3]. When these data are combined with the significantly lower concentration of PON3 in serum/plasma compared to PON1 [44], it suggests that PON3’s impact on HDL lactonase activity may also be limited. Lactonase activity is believed to be the key driver of the various well-recognized beneficial actions of PON1, besides its antioxidative protection of LDL [45]. These include: (1) protecting endothelial cells and macrophages from oxidation, either through direct action on membrane lipolactones or by downregulating intracellular production of reactive species [46]; (2) decreasing monocyte chemotaxis and adhesion to endothelial cells [46]; (3) suppressing the secretion of pro-inflammatory cytokines [47]; (4) inhibiting monocyte-to-macrophage differentiation [48]; (5) increasing the activity of endothelial nitric oxide synthase (eNOS), thereby enhancing the production of the vasodilator nitric oxide [48]; (6) impacting cholesterol homeostasis by upregulating the efflux of cholesterol from macrophages and foam cells [49].

Differences between PON1 and PON3 also emerged from the analysis of their correlation with HDL-C and MPO.

In line with previous reports, arylesterase activity was found to be associated with serum HDL-C concentration [16,50]. At first glance, this correlation might seem weaker than expected, given that HDL is the main carrier of PON1. However, it is important to consider that HDL-C (i.e., the cholesterol concentration in HDL) is not a reliable surrogate for HDL particle concentration. Additionally, it has become evident that PON1 is predominantly bound to a specific subtype of HDL (small dense HDL), which constitutes only a minor and variable fraction of the total circulating HDL [51,52]. Considering that the concentration of PON3 is almost 100 times lower than that of PON1, an even smaller fraction of PON3 could be associated with the small dense HDL, and this could explain the lack of correlation between this protein and HDL-C.

Myeloperoxidase (MPO) generates highly reactive species, such as hypochlorous acid, in collaboration with other pro-oxidant enzymes [26,34]. These reactive species indiscriminately target and damage both non-self and self-biological entities, with HDL and LDL being primary targets [26,53]. Under inflammatory conditions, MPO is secreted in abnormal amounts from neutrophils and physically binds to HDL [26,54]. This binding leads to the oxidative modification of various HDL components, particularly PON1 and APOA1, thereby rendering HDL dysfunctional and less effective in protecting LDL from oxidation [24,26].

Despite in vitro evidence suggesting a reciprocal regulation between PON1 and MPO, we were not surprised by the observed lack of correlation between these two proteins (see Figure 5B). It is plausible that MPO’s activity interferes more with the native lactonase function of PON1 rather than its promiscuous arylesterase activity. More surprising, however, was the relatively strong positive correlation found between MPO and PON3. This finding suggests that PON3 might be preferentially associated with the fraction of HDL enriched with MPO, although there was no observed inhibitory effect of PON3 on this heme protein (MPO-specific activity did not correlate with PON3).

The hypothesized preferential placement of PON3 in dysfunctional HDL aligns with observational studies on diseases characterized by high concentrations of dysfunctional HDL. For instance, Rull et al. [44] found that serum PON3 levels were increased in subjects with peripheral artery disease or coronary artery disease, conditions marked by elevated levels of dysfunctional HDL, compared to healthy controls. Elevated serum PON3 levels were also observed in patients with HIV-1 [55], a condition associated with macrophage activation, cardiovascular disease, and direct upregulation of T-cell activation in vitro, a hallmark of this infectious disease [56,57].

We must also recognize some important limitations and strengths of this study. First, the cross-sectional design limits our ability to determine a cause-and-effect relationship between arylesterase activity, PON3, and the development of MCI or AD. Second, it is possible that biases or unmeasured variables may have influenced our findings. However, we accounted for several potential confounding factors (e.g., age, gender, etc.) known to be associated with PON1, PON3, and neurodegenerative diseases, and the significant results observed were independent of these factors. Third, the participants in this study were not assessed for CSF biomarkers of AD, meaning the possibility of misclassification cannot be entirely dismissed. We acknowledge that the lack of these data impacts the scientific significance of our findings. Fourth, the interpretation of the findings related to PON3 is constrained by the limited understanding of this protein’s function, which may make the conclusions speculative. However, this also represents a key strength of the study, as we are the first to investigate serum PON3 in the context of Alzheimer’s disease and its association with MPO. Fifth, the lack of data on PON1 concentration determined through ELISA prevented us from comparing the relative levels of PON1 and PON3 and, therefore, from relating their relative variation in the pathological context of our study. Although PON1 activity is a good surrogate for its concentration measurement given the marginal influence of genetic polymorphisms on the activity, its specific activity varies depending on pathological conditions and comorbidities [58]. Therefore, it was not possible with our data to estimate the possible range of PON1 concentrations present in our population. Nonetheless, published studies suggest that the concentration of PON3 is 50–100 times lower than that of PON1 [59].

## 5. Conclusions

In conclusion, our study is the first to explore serum PON3 in the context of Alzheimer’s disease (AD) and mild cognitive impairment (MCI) and to compare its pattern with PON1-arylesterase activity. We found that while serum arylesterase activity significantly decreased in both MCI and AD compared to controls, PON3 levels remained unchanged. Another noteworthy finding was the strong correlation between PON3, but not arylesterase activity, and the pro-oxidant, pro-atherogenic MPO, which may suggest a preferential physical association of PON3 with dysfunctional HDL. Further research is warranted to clarify the role of PON3 in HDL functionality and its potential impact on diseases characterized by oxidative stress and inflammation.

## Figures and Tables

**Table 1:** Principal characteristics of the sample according to the diagnosis.

	CONTROLS (*n* = 79)	MCI (*n*= 100)	AD (*n*= 99)
Age (years)	76 ± 6	78 ± 5 ^a^	78 ± 5 ^a^
Female gender (%)	64	57	75 ^a,b^
Formal Education (years)	7 (5–12)	5 (4–8) ^a^	5 (4–8) ^a^
MMSE score (/30)	27 (26–30)	25 (23–26) ^a^	20 (18–23) ^a,b^
- Comorbidities			
Hypertension (%)	55	68 ^a^	62
Diabetes (%)	7	10	8
CHD (%)	7	14	10
Stroke (%)	3	4	2
Smoking (%)	8	10	10
- Metabolic markers *			
HDL-C, mg/dL	59 ± 17	60 ± 17	62 ± 16
LDL-C, mg/dL	129 ± 38	119 ± 35	125 ± 35
Tryglycerides, mg/dL	115 ± 41	112 ± 51	107 ± 60
Total Cholesterol, mg/dL	210 ± 44	203 ± 42	209 ± 39
Glucose, mg/dL	103 ± 29	104 ± 32	95 ± 24

Variables are expressed as mean ± SD or median (interquartile range) when normally and NON-normally distributed, respectively. ^a^ *p* < 0.05 vs. CONTROLS; ^b^ *p* < 0.05 vs. MCI. Abbreviations: MCI, Mild Cognitive Impairment. * Lipid profile was assessed in 75 controls, 97 MCI and 99 AD. SD, standard deviation; AD, Alzheimer’s disease; CHD, Coronary Heart Disease; MMSE, Mini Mental State Examination; HDL-C, High-density lipoprotein-cholesterol; LDL-C, Low-density lipoprotein-cholesterol.

## Data Availability

The data presented in this study are available on request from the corresponding author.

## Supplementary Materials

The following supporting information can be downloaded at: https://www.mdpi.com/article/10.3390/antiox13101216/s1. Table S1: MPO protein concentration and actvity in Controls, MCI and AD.
