# Supplementary material for: PON1 and PON3 in Alzheimer’s Disease: Similar Functions but Different Roles
Source: Antioxidants (Basel). 2024 Oct 10;13(10):1216. doi: 10.3390/antiox13101216 (PMC11505261; doi:10.3390/antiox13101216)
Supplement: Supplementary file 1 [file antioxidants-13-01216-s001.zip › antioxidants-3226649-supplementary.pdf]

Supplementary Table S1: MPO protein concentration and activity in Controls, MCI and AD

	CONTROLS (n = 50)	MCI (n = 28)	AD (n = 35)
**MPO, ng/mL**	111 (74–144)	99 (54–182)	89 (66–142)
**MPO, U/L**	0.033 (0.013–0.062)	0.010 (0.041–0.028)	0.035 (0.003–0.067)

Variables are expressed median (interquartile range)

Abbreviations: AD, Alzheimer's disease; MCI, Mild Cognitive Impairment; MPO, Myeloperoxidase
